# Prevalence of Amyloidosis in Korea

**DOI:** 10.1186/s13023-017-0705-2

**Published:** 2017-09-06

**Authors:** Su Ra Seo, Shin Yi Jang, Ga Yeon Lee, Bareun Choi, Heeran Chun, Eun Jeong Cho, Sung-il Cho

**Affiliations:** 1 Graduate School of Public Health, Seoul National University, Seoul, 08826 Republic of Korea; 2 The National Health Insurance Service, Wonju, 25234 Republic of Korea; 3 Division of Cardiology, Department of Medicine, Imaging Center, Heart Vascular Stroke Institute, Samsung Medical Center, Sungkyunkwan University School of Medicine, Seoul, 06351 Republic of Korea; 4 Namyangju Poongyang Public Health Center, Namyangju, 12066 Republic of Korea; 5 Department of Health Administration, Jungwon University, Goesan, 28024 Republic of Korea; 6 Division of Cardiology, Department of Internal Medicine, Cardiology Clinic, National Cancer Center, Goyang, 10408 Republic of Korea

**Keywords:** Prevalence, Amyloidosis, Korea

## Abstract

**Background:**

The aim of this study was to assess in amyloidosis prevalence in Korea between 2006 and 2015.

**Methods:**

Primary diagnoses related to amyloidosis, regardless of subtype, were collected from the Korean National Health Insurance Service from 2006 through 2015.

**Results:**

Overall, the age-standardized prevalence of amyloidosis was 0.93 (95% confidence interval (CI) 0.81, 1.04) persons per 100,000 persons in 2006 and 1.91 (95% CI 1.78, 2.05) persons per 100,000 persons in 2015. This included an increase from 0.43 (95% CI 0.35, 0.51) to 1.04 (95% CI 0.94, 1.14) persons per 100,000 persons in men and from 0.49 (95% CI 0.40, 0.57) to 0.87 (95% CI 0.77, 0.96) persons per 100,000 persons in women. In particular, the age-standardized prevalence of amyloidosis showed a greater increase in patients aged 65 years or older and in patients aged 45–64 years than in patients aged 20–44 years, for both men and women.

**Conclusions:**

The overall age-standardized prevalence of amyloidosis was approximately 2 persons per 100,000 persons in 2015. The overall age-standardized prevalence of amyloidosis increased between 2006 and 2015, especially in individuals aged 45–64 and older than 65 years.

**Electronic supplementary material:**

The online version of this article (10.1186/s13023-017-0705-2) contains supplementary material, which is available to authorized users.

## Background

Amyloidosis is a rare disease that can cause systemic dysfunction and abnormal fibrillar protein deposits in the extracellular space, resulting in impaired function of various organs. The kidney and heart are most commonly involved, but the nerves and gastrointestinal tract can also be affected [1–4]. Cardiac involvement is the major determinant of prognosis in amyloidosis patients [5]. The subtypes of amyloidosis are determined by the biochemical properties of the progenitor proteins that form the deposited fibers. These subtypes require very different treatment methods [6]. Therefore, determining the correct amyloidosis subtype is important. Diagnosis of amyloidosis requires tissue biopsy, which is assessed for evidence of characteristic amyloid deposits using treatment with various stains [7]. The major systemic types of amyloidosis are AL, which is associated with light chain-producing plasma cell dyscrasia; AA, which is associated with longstanding inflammation; wild-type ATTR, which is associated with normal transthyretin and old age; and hereditary ATTR, which is associated with a transthyretin mutation [1]. Half of patients with familial amyloidosis have been shown to have no family history of the disease [8]. If amyloidosis is not treated properly, it has a negative prognosis such as a low survival rate. We analyzed changes in the age-adjusted prevalence of amyloidosis in Korea from 2006 through 2015. We hope that these basic data will be used to improve the quality of life and to extend lives of amyloidosis patients through successful intervention following earlier diagnosis [9].

## Methods

### Study population

Data were collected from Korean National Health Insurance (KNHI) benefit records from 2006 until 2015. The KNHI provides compulsory social insurance in the form of employee medical insurance and self-employed medical insurance that covers approximately 98% of the people living in the country. Enrollment in KNHI is enforced for all citizens, and enrollees are obligated to pay insurance premiums assessed based on income level. Regardless of the level of insurance premiums, KNHI benefits are paid evenly under the relevant laws and regulations. Medical Aid targets low-income recipients under the National Basic Livelihood Security Act and covers about 2% of the total population [10]. We excluded records from the Medical Aid branch of the National Health Insurance Service. We analyzed records of primary diagnoses related to amyloidosis using the following 10th revision of the International Statistical Classification of Diseases and Related Health Problems (ICD 10) codes regardless of subtype: E85.0 Non-neuropathic heredofamilial amyloidosis; E85.1 Neuropathic heredofamilial amyloidosis; E85.2 Heredofamilial amyloidosis, unspecified; E85.3 Secondary systemic amyloidosis; E85.4 Organ-limited amyloidosis; E85.8 Other amyloidosis; and E85.9 Amyloidosis, unspecified.

### Statistical methods

The age-standardized prevalence of amyloidosis was calculated with the direct method [11] using the beneficiaries of health insurance from the Korean National Health Insurance Statistical Yearbook from 2006 through 2015 [12–21] and the estimated Korean population in 2015 [22] as a reference [23, 24].

## Results

Overall, the age-standardized prevalence of amyloidosis was 0.93 persons per 100,000 persons in 2006 and 1.91 persons per 100,000 persons in 2015. This included an increase from 0.43 to 1.04 persons per 100,000 persons in men and from 0.49 to 0.87 persons per 100,000 persons in women. For males in 2006, the prevalence of amyloidosis per 100,000 persons was 0.14 persons aged 20–44 years, 0.37 persons aged 45–64 years, and 1.06 persons older than 65 years. For males in 2015, the prevalence of amyloidosis per 100,000 persons was 0.29 persons aged 20–44 years, 1.18 persons aged 45–64 years, and 2.90 persons older than 65 years. For females in 2006, the prevalence of amyloidosis per 100,000 persons was 0.13 persons aged 20–44 years, 0.58 persons aged 45–64 years, and 0.97 persons older than 65 years. For females in 2015, the prevalence of amyloidosis per 100,000 persons was 0.23 persons aged 20–44 years, 1.16 persons aged 45–64 years, and 2.02 persons older than 65 years (Table 1).

**Table 1:** Age-adjusted cumulative prevalence ^a^ of amyloidosis, both overall and by gender (per 100,000 persons), along with 95% confidence intervals (CI)

Variable	2006		2007		2008		2009		2010	
	n	Prevalence (95% CI)	n	Prevalence (95% CI)	n	Prevalence (95% CI)	n	Prevalence (95% CI)	n	Prevalence (95% CI)
All	253	0.93 (0.81, 1.04)	334	1.17 (1.04, 1.30)	376	1.26 (1.13, 1.39)	414	1.31 (1.18, 1.44)	424	1.30 (1.18, 1.43)
20–44 years old	58	0.28 (0.20, 0.35)	60	0.29 (0.21, 0.36)	71	0.35 (0.27, 0.43)	87	0.43 (0.34, 0.53)	65	0.32 (0.24, 0.40)
45–64 years old	111	0.97 (0.79, 1.15)	164	1.38 (1.17, 1.59)	169	1.37 (1.16, 1.58)	192	1.50 (1.29, 1.73)	220	1.66 (1.44, 1.89)
Over 65 years	84	2.05 (1.61, 2.49)	110	2.49 (2.02, 2.96)	136	2.95 (2.45, 3.45)	135	2.78 (2.31, 3.25)	139	2.78 (2.32, 3.24)
Men	118	0.43 (0.35, 0.51)	172	0.60 (0.51, 0.69)	187	0.62 (0.53, 0.71)	231	0.73 (0.63, 0.83)	230	0.71 (0.61, 0.80)
20–44 years old	30	0.14 (0.09, 0.19)	31	0.14 (0.09, 0.20)	39	0.19 (0.13, 0.25)	48	0.23 (0.16, 0.30)	38	0.18 (0.12, 0.25)
45–64 years old	44	0.37 (0.26, 0.49)	82	0.69 (0.54, 0.84)	81	0.65 (0.51, 0.80)	104	0.81 (0.66, 0.97)	108	0.81 (0.66, 0.97)
Over 65 years	44	1.06 (0.74, 1.38)	59	1.33 (0.99, 1.68)	67	1.44 (1.09, 1.79)	79	1.62 (1.26, 1.98)	84	1.67 (1.31, 2.03)
Women	135	0.49 (0.40, 0.57)	162	0.56 (0.47, 0.65)	189	0.63 (0.54, 0.73)	183	0.57 (0.49, 0.66)	194	0.59 (0.51, 0.68)
20–44 years old	28	0.13 (0.08, 0.18)	29	0.14 (0.09, 0.19)	32	0.15 (0.09, 0.21)	39	0.19 (0.13, 0.25)	27	0.13 (0.08, 0.18)
45–64 years old	67	0.58 (0.44, 0.72)	82	0.69 (0.54, 0.84)	88	0.71 (0.56, 0.86)	88	0.69 (0.54, 0.83)	112	0.84 (0.68, 1.00)
Over 65 years	40	0.97 (0.66, 1.27)	51	1.15 (0.83, 1.47)	69	1.49 (1.13, 1.84)	56	1.15 (0.85, 1.46)	55	1.09 (0.80, 1.38)
Variable	2011		2012		2013		2014		2015	
	n	Prevalence (95% CI)	n	Prevalence (95% CI)	n	Prevalence (95% CI)	n	Prevalence (95% CI)	n	Prevalence (95% CI)
All	454	1.35 (1.22, 1.47)	577	1.64 (1.50, 1.77)	638	1.71 (1.58, 1.85)	725	1.86 (1.73, 2.00)	771	1.91 (1.78, 2.05)
20–44 years old	63	0.32 (0.24, 0.40)	76	0.38 (0.30, 0.47)	90	0.46 (0.36, 0.56)	104	0.54 (0.43, 0.64)	103	0.54 (0.43, 0.64)
45–64 years old	218	1.59 (1.38, 1.80)	269	1.92 (1.69, 2.15)	320	2.21 (1.97, 2.45)	343	2.30 (2.05, 2.54)	361	2.35 (2.11, 2.59)
Over 65 years	173	3.33 (2.83, 3.83)	232	4.23 (3.68, 4.77)	228	3.95 (3.44, 4.47)	278	4.62 (4.08, 5.17)	307	4.93 (4.38, 5.48)
Men	235	0.69 (0.60, 0.78)	305	0.86 (0.76, 0.95)	355	0.95 (0.85, 1.05)	372	0.95 (0.86, 1.05)	420	1.04 (0.94, 1.14)
20–44 years old	34	0.17 (0.11, 0.23)	48	0.24 (0.17, 0.31)	52	0.26 (0.19, 0.33)	57	0.29 (0.21, 0.37)	57	0.29 (0.22, 0.37)
45–64 years old	106	0.77 (0.62, 0.92)	138	0.98 (0.81, 1.14)	164	1.13 (0.95, 1.30)	169	1.13 (0.95, 1.30)	182	1.18 (1.01, 1.35)
Over 65 years	95	1.82 (1.45, 2.19)	119	2.16 (1.77, 2.55)	139	2.42 (2.01, 2.82)	146	2.42 (2.02, 2.81)	181	2.90 (2.48, 3.33)
Women	219	0.64 (0.56, 0.73)	272	0.77 (0.68, 0.86)	283	0.75 (0.66, 0.84)	353	0.90 (0.81, 1.00)	351	0.87 (0.77, 0.96)
20–44 years old	29	0.14 (0.09, 0.19)	28	0.13 (0.08, 0.19)	38	0.19 (0.13, 0.25)	47	0.24 (0.17, 0.31)	46	0.23 (0.16, 0.30)
45–64 years old	112	0.81 (0.66, 0.97)	131	0.93 (0.77, 1.09)	156	1.07 (0.90, 1.24)	174	1.16 (0.99, 1.33)	179	1.16 (0.99, 1.33)
Over 65 years	78	1.49 (1.15, 1.82)	113	2.05 (1.67, 2.43)	89	1.53 (1.21, 1.85)	132	2.19 (1.81, 2.56)	126	2.02 (1.67, 2.37)

^a^Age-standardized prevalence rates of amyloidosis were calculated using age groups following the direct method using the estimated Korean population in 2015 as a reference

## Discussion

Our results demonstrate that the overall age-standardized prevalence of amyloidosis in 2015 was approximately 2 persons per 100,000 persons in Korea. Also, the overall age-standardized prevalence of amyloidosis increased between 2006 and 2015 in patients over 45 years of age. Few studies have reported the overall age-standardized prevalence of amyloidosis. Therefore, the results in this study cannot be compared to those of other geographic or ethnic groups. Nevertheless, the results of the present study are similar to those of earlier studies. The estimated minimum prevalence of systemic amyloidosis in the United Kingdom is 20 persons per one million [25]. In our study, the age-standardized prevalence of amyloidosis showed a greater increase in patients aged 65 years or older and those aged 45–64 years than it did in patients aged 20–44 years for both men and women. Thus, our results indicate that amyloidosis tends to affect older people. This result is consistent with previous reports from the National Organization for Rare Disorders. Approximately 4000 new persons are diagnosed with AL amyloidosis annually in the United States (U.S.), with symptoms typically diagnosed at age 50–65. Moreover, familial amyloidosis, caused by a transthyretin mutation occurs in approximately 1 of every 100,000 Caucasians in the U.S. and more commonly among African Americans (approximately 4% in that population). The symptoms usually begin between 40 and 65 years of age [26].

To address potential concerns about the accuracy of the data recorded during the study period, we obtained additional data consisting of main diagnoses that corresponded to National Health Insurance expanded benefit coverage for a rare incurable disease (expanded benefit coverage) as the main diagnosis from 2009. In order to be qualified for the expanded benefit coverage, the amyloidosis should be designated as primary diagnoses. The expanded benefit coverage special number is V121. The expanded benefit coverage policy is applied to patients who have paid 10% of the total amount of the medical treatment cost for either outpatient or inpatient care for 5 years from the registration date. That is, the National Health Insurance Service renews the expanded benefit coverage qualifications every five years. We also found similar distributions of amyloidosis in the 2015 data from the National Health Insurance Service (*n* = 771) and the 2011 to 2015 National Health Insurance Service expanded benefit coverage (*n* = 749) (Additional file 1: Table S1).

For the further analysis, we calculated the age-standardized prevalence using the direct method with the World Health Organization (WHO) standard population in 2000–2025 [27] as a reference (Additional file 1: Table S2). Overall, the age-standardized prevalence of amyloidosis was 6.92 persons per 100,000 persons in 2006 and 21.1 persons per 100,000 persons in 2015. In other words, using the WHO standard population in 2000–2025 as the reference, the age-standardized prevalence of amyloidosis has tripled during the past decade.

### Study limitations

Our study has several limitations. First, our data included only major amyloidosis and did not consider other conditions, such as amyloidosis subtype. Furthermore, we used the primary diagnosis based on signs and symptoms, which might differ from the final diagnosis. Thus, the prevalence of amyloidosis in this study might be under- or overestimated. To address this limitation, we compared our results with an analysis of expanded benefit coverage data. However, further collection of nationwide registry data with definite inclusion criteria using specific organ biopsies will be required to confirm amyloidosis. We also carried out an additional analysis for the percentage of each subtype of amyloidosis by ICD 10 code in 2015: E85.0 Non-neuropathic heredofamilial amyloidosis (1.48%); E85.1 Neuropathic heredofamilial amyloidosis (0.94%); E85.2 Heredofamilial amyloidosis, unspecified (1.48%); E85.3 Secondary systemic amyloidosis (0.0%); E85.4 Organ-limited amyloidosis (25.9%); E85.8 Other amyloidosis (10.7%); and E85.9 Amyloidosis, unspecified (59.5%). Second, the National Health Insurance benefit records might have missed potential patients with amyloidosis who did not use medical services, paid for their own medical expenses, or received Medical Aid. Therefore, because our data indicate that the prevalence of amyloidosis increases with age, a hospital-based amyloidosis registry is needed in Korea. Third, we considered only age, gender, and year. Our data did not include risk factors for chronic heart or kidney disease, socioeconomic position, or health behavior factors, so we could not adjust the prevalence of amyloidosis for several possible confounding variables. Therefore, a follow-up study is needed to determine the long-term survival rate of patients with amyloidosis after adjusting for confounding variables.

## Conclusions

We found that the overall age-standardized prevalence of amyloidosis was approximately 2 persons per 100,000 persons in 2015. Our results might have important clinical and public health implications because subjects with amyloidosis tend to be elderly.

## Additional file

Additional file 1:
**Table S1.** New patients with amyloidosis (special numbers for expanded benefit coverage V121) according to National Health Insurance expanded benefit coverage by year. **Table S2.** Age-adjusted cumulative prevalence ^a^ of amyloidosis, both overall and by gender (per 100,000 persons), along with 95% confidence intervals (CI) using World Health Organization standard population in 2000. (DOC 85 kb)

## Abbreviations

expanded benefit coverage: National Health Insurance expanded benefit coverage for a rare incurable disease
US: United States
WHO: World Health Organization
