# Supplementary material for: Prevalence of Amyloidosis in Korea
Source: Orphanet J Rare Dis. 2017 Sep 6;12:152. doi: 10.1186/s13023-017-0705-2 (PMC5588743; doi:10.1186/s13023-017-0705-2)
Supplement: Additional file 1: — Table S1. New patients with amyloidosis (special numbers for expanded benefit coverage V121) according to National Health Insurance expanded benefit coverage by year. Table S2. Age-adjusted cumulative prevalence a of amyloidosis, both overall and by gender (per 100,000 persons), along with 95% confidence intervals (CI) using World Health Organization standard population in 2000. (DOC 85 kb) [file 13023_2017_705_MOESM1_ESM.doc]

Additional file 1: Table S1. New patients with amyloidosis (special numbers for expanded benefit coverage V121) according to National Health Insurance expanded benefit coverage by year

| Year | Number of persons |
| --- | --- |
| 2009 | 182 |
| 2010 | 104 |
| 2011 | 100 |
| 2012 | 131 |
| 2013 | 126 |
| 2014 | 202 |
| 2015 | 190 |
| 2016 | 201 |

Table S2. Age-adjusted cumulative prevalence ^a^ of amyloidosis, both overall and by gender (per 100,000 persons), along with 95% confidence intervals (CI)

| Variable | 2006 | | 2007 | | 2008 | | 2009 | | 2010 | |
| --- | --- | --- | --- | --- | --- | --- | --- | --- | --- | --- |
|  | n | Prevalence  (95% CI) | n | Prevalence  (95% CI) | n | Prevalence  (95% CI) | n | Prevalence  (95% CI) | n | Prevalence  (95% CI) |
| All | 253 | 6.92 (6.07, 7.78) | 334 | 9.14 (8.16, 10.1) | 376 | 10.2 (9.25, 11.3) | 414 | 11.3 (10.2, 12.4) | 424 | 11.6 (10.5, 12.7) |
| 20–44 years old | 58 | 4.21 (3.13, 5.30) | 60 | 4.36 (3.25, 5.46) | 71 | 5.16 (3.96, 6.36) | 87 | 6.32 (4.99, 7.65) | 65 | 4.72 (3.57, 5.87) |
| 45–64 years old | 111 | 9.27 (7.54, 11.0) | 164 | 13.7 (11.6, 15.8) | 169 | 14.1 (11.9, 16.2) | 192 | 16.0 (13.7, 18.3) | 220 | 18.3 (15.9, 20.8) |
| Over 65 years | 84 | 7.77 (6.10, 9.43) | 110 | 10.1 (8.27, 12.0) | 136 | 12.5 (10.4, 14.6) | 135 | 12.4 (10.3, 14.5) | 139 | 12.8 (10.7, 14.9) |
| Men | 118 | 3.23 (2.64, 3.81) | 172 | 4.70 (4.00, 5.41_ | 187 | 5.11 (4.38, 5.85) | 231 | 6.32 (5.50, 7.14) | 230 | 6.29 (5.48, 7.11) |
| 20–44 years old | 30 | 2.18 (1.40, 2.96) | 31 | 2.25 (1.46, 3.04) | 39 | 2.83 (1.94, 3.72) | 48 | 3.49 (2.50, 4.47) | 38 | 2.76 (1.88, 3.64) |
| 45–64 years old | 44 | 3.67 (2.59, 4.76) | 82 | 6.85 (5.36, 8.33) | 81 | 6.76 (5.29, 8.24) | 104 | 8.69 (7.02, 10.3) | 108 | 9.02 (7.32, 10.7) |
| Over 65 years | 44 | 4.07 (2.86, 5.27) | 59 | 5.45 (4.06, 6.85) | 67 | 6.19 (4.71, 7.68) | 79 | 7.30 (5.69, 8.92) | 84 | 7.77 (6.10, 9.43) |
| Women | 135 | 3.69 (3.07, 4.31) | 162 | 4.43 (3.75, 5.11) | 189 | 5.17 (4.43, 5.91) | 183 | 5.01 (4.28, 5.73) | 194 | 5.31 (4.56, 6.05) |
| 20–44 years old | 28 | 2.03 (1.28, 2.79) | 29 | 2.10 (1.34, 2.87) | 32 | 2.32 (1.52, 3.13) | 39 | 2.83 (1.94, 3.72) | 27 | 1.96 (1.22, 2.70) |
| 45–64 years old | 67 | 5.59 (4.25, 6.93) | 82 | 6.85 (5.36, 8.33) | 88 | 7.35 (5.81, 8.88) | 88 | 7.35 (5.81, 8.88) | 112 | 9.35 (7.62, 11.0) |
| Over 65 years | 40 | 3.70 (2.55, 4.84) | 51 | 4.71 (3.42, 6.01) | 69 | 6.38 (4.87, 7.89) | 56 | 5.18 (3.82, 6.53) | 55 | 5.08 (3.74, 6.43) |
|  |  |  |  |  |  |  |  |  |  |  |
| Variable | 2011 | | 2012 | | 2013 | | 2014 | | 2015 | |
|  | n | Prevalence  (95% CI) | n | Prevalence  (95% CI) | n | Prevalence  (95% CI) | n | Prevalence  (95% CI) | n | Prevalence  (95% CI) |
| All | 454 | 12.4 (11.2, 13.5) | 577 | 15.7 (14.5, 17.0) | 638 | 17.4 (16.1, 18.8) | 725 | 19.8 (18.4, 21.2) | 771 | 21.1 (19.6, 22.5) |
| 20–44 years old | 63 | 4.58 (3.45, 5.71) | 76 | 5.52 (4.28, 6.77) | 90 | 6.54 (5.19, 7.89) | 104 | 7.56 (6.11, 9.01) | 103 | 7.49 (6.04, 8.93) |
| 45–64 years old | 218 | 18.2 (15.7, 20.6) | 269 | 22.4 (19.7, 25.1) | 320 | 26.7 (23.8, 29.6) | 343 | 28.6 (25.6, 31.6) | 361 | 30.1 (27.0, 33.2) |
| Over 65 years | 173 | 16.0 (13.6, 18.3) | 232 | 21.4 (18.7, 24.2) | 228 | 21.0 (18.3, 23.8) | 278 | 25.7 (22.6, 28.7) | 307 | 28.4 (25.2, 31.5) |
| Men | 235 | 6.43 (5.61, 7.25) | 305 | 8.35 (7.41, 9.28) | 355 | 9.71 (8.70, 10.7) | 372 | 10.1 (9.15, 11.2) | 420 | 11.4 (10.3, 12.5) |
| 20–44 years old | 34 | 2.47 (1.64, 3.30) | 48 | 3.49 (2.50, 4.47) | 52 | 3.78 (2.75, 4.81) | 57 | 4.14 (3.06, 5.22) | 57 | 4.14 (3.06, 5.22) |
| 45–64 years old | 106 | 8.85 (7.17, 10.5) | 138 | 11.5 (9.60, 13.4) | 164 | 13.7 (11.6, 15.8) | 169 | 14.1 (11.9, 16.2) | 182 | 15.2 (12.9, 17.4) |
| Over 65 years | 95 | 8.78 (7.02, 10.5) | 119 | 11.0 (9.03, 12.9) | 139 | 12.8 (10.7, 14.9) | 146 | 13.5 (11.3, 15.6) | 181 | 16.7 (14.3, 19.1) |
| Women | 219 | 5.99 (5.20, 6.79) | 272 | 7.44 (6.56, 8.33) | 283 | 7.74 (6.84 (8.65) | 353 | 9.66 (8.65, 10.6) | 351 | 9.61 (8.60, 10.6) |
| 20–44 years old | 29 | 2.10 (1.34, 2.87) | 28 | 2.03 (1.28, 2.79) | 38 | 2.76 (1.88, 3.64) | 47 | 3.41 (2.44, 4.39) | 46 | 3.34 (2.37, 4.31) |
| 45–64 years old | 112 | 9.35 (7.62, 11.0) | 131 | 10.9 (9.07, 12.8) | 156 | 13.0 (10.9, 15.0) | 174 | 14.5 (12.3, 16.7) | 179 | 14.9 (12.7, 17.1) |
| Over 65 years | 78 | 7.21 (5.61, 8.81) | 113 | 10.4 (8.52, 12.3) | 89 | 8.23 (6.52, 9.94) | 132 | 12.2 (10.1, 14.2) | 126 | 11.6 (9.62, 13.6) |

a Age-standardized prevalence rates of amyloidosis were calculated using age groups following the direct method using World Health Organization standard population in 2000 as a reference.
